# Responsible Open Science: Moving towards an Ethics of Environmental Sustainability

**DOI:** 10.3390/publications8040054

**Published:** 2020-12-11

**Authors:** Gabrielle Samuel, Federica Lucivero

**Affiliations:** 1 Department of Global Health and Social Medicine, King’s College London, London WC2B 4BG, UK; 2 Ethox Centre, Wellcome Centre for Ethics and Humanities, University of Oxford, Oxford OX3 7LF, UK

**Keywords:** open science, digital sustainability, environmental impacts, responsible research, ethics

## Abstract

The integration of open science as a key pillar of responsible research and innovation has led it to become a hallmark of responsible research. However, ethical, social and regulatory challenges still remain about the implementation of an internationally- and multi-sector-recognised open science framework. In this Commentary, we discuss one important specific challenge that has received little ethical and sociological attention in the open science literature: the environmental impact of the digital infrastructure that enables open science. We start from the premise that a move towards an environmentally sustainable open science is a shared and valuable goal, and discuss two challenges that we foresee with relation to this. The first relates to questions about how to define what environmentally sustainable open science means and how to change current practices accordingly. The second relates to the infrastructure needed to enact environmentally sustainable open science ethical and social responsibilities through the open science ethics ecosystem. We argue that there are various ethical obstacles regarding how to responsibly balance any environmental impacts against the social value of open science, and how much one should be prioritised over the other. We call for all actors of the open science ethics ecosystem to engage in discussions about how to move towards open data and science initiatives that take into account the environmental impact of data and digital infrastructures. Furthermore, we call for ethics governance frameworks or policy-inscribed standards of practice to assist with this decision-making.

## 1 Introduction

The integration of open science as a key pillar of responsible research and innovation has led it to become a hallmark of responsible research in higher education institutions and other governmental and non-governmental research organisations. A range of researchers, institutions and professional bodies now position themselves in alignment with the key principles of this initiative, and its enshrined values are becoming increasingly widespread throughout the research infrastructure [1–3]. While a range of sociological and ethical critiques about the political intentions of the open science initiative have emerged [4,5], its ambition is generally considered positive, acting to improve knowledge circulation and innovation, promote accessibility and replication, and provide increased research and researcher integrity and accountability [4,6,7]. Such goals are particularly important given the increasing reliance of research on mega datasets and artificial intelligence: as we move towards the collection of vast amounts of ‘big data’, accessibility to datasets and software can allow data re-use, thereby strengthening research efficiency and value. Ethical, social and regulatory challenges still remain about the implementation of an internationally- and multi-sector-recognised open science framework, many of which are discussed in other articles of this special issue (including issues relating to the value of openness and transparency, incentivisation, responsibility and publishing ethics). However, much policy and scholarly work is increasingly being invested into mitigating (some of) these barriers (for example, see [8–10]).

In this commentary, we discuss one important specific challenge that has received little ethical and sociological attention in the open science literature: the environmental impact of the digital infrastructure that enables it. The underlying premise of open science relies on data infrastructures that enable the storage of, and access to large amounts of data. By data infrastructures, we are referring to the data, data storage centres, data processing, and data platforms, services and tools etc. that make up the entire data lifecycle. Together, these have considerable environmental impacts. They include heavy carbon dioxide emissions linked to the energy required to generate, store and process large amounts of data; the impact on the material environment (e.g., where data centres are constructed); and the use of unsustainable practices for both extracting minerals for technological components, as well as e-waste disposal [11]. These are important concerns: to meet the Paris targets and limit earth temperature increase to 1.5 degrees Celsius, carbon emissions from all sectors including information and communication technologies (ICT) need to drop significantly [12,13]. While likely improvements in energy efficiency and the move to renewable energy will no doubt relieve at least some of these concerns, renewable energy is not a panacea to climate issues [11]. Furthermore, the pace of data-driven innovation in the ICT sector raises concerns that it could potentially dominate the world’s renewable energy sources, leading to increases in carbon emissions when other sectors are decreasing their energy use.

The environmental impacts of data infrastructures have long been known and are widely acknowledged in the ICT industry and environmental sciences [13–15]. Several published reports have emphasised the need for policymakers to incentivise companies and data centres to reduce their carbon footprint, and recommendations to improve data centre energy efficiency have been listed by environmental and scientific agencies, as well as by the wider scholarly and policy community [16,17] (see also [18–21]). However, such discussions have featured little, if at all, in the literature on open science. With the continuing need for data infrastructures to support an increasing rise in the number of open science projects (including increases in storage capacities, and processing as data is re-used), there is an ethical and social responsibility for actors operating in the field to consider the environmental impacts of open science. If we assume that the move towards an environmentally sustainable open science should be seen as a shared and valuable goal (allowing that this premise in itself requires further consideration), we need to reflect on existing challenges to achieve such a goal.

This is not to say that open science is unique in terms of needing to focus on issues of environmental sustainability; data repositories and similar infrastructures would be in the landscape even without open science. Further, while open science might raise additional costs in terms of storing data for further re-use, as well as creating opportunities for third parties to do more data processing (if data is there it is more likely to be duplicated, used, or processed for both valuable, but perhaps also less valuable goals (see below)), the tenets of open science do not necessarily result in specific challenges that are not already present in ‘closed’ science or ICT in general; and large-scale data collection with power-hungry instruments is related to both open and closed science. As such, the points we make in this commentary are applicable to both open and ‘closed’ science, and in fact, we view the need for an environmental sustainability of open science to be just one aspect of such a requirement for all data science.

We foresee a number of challenges related to moving towards an environmentally sustainable open science. In the remainder of the commentary, we focus on two such challenges that specifically arise from the perspective of those individuals who are considering opening their data sets (researchers, curators, etc.). The first challenge relates to how to define what environmentally sustainable open science means and how to change current practices accordingly given the lack of evidence and best practice. The second relates to the infrastructure needed to enact environmentally sustainable open science ethical and social responsibilities through the open science ethics ecosystem.

## 2 Difficulties Assessing Open Science Environmental Impacts

In other sectors, scholars and policymakers are beginning to grapple with questions about how to change current practices to make them more environmentally sustainable, and these individuals have made some progress. Their first step is to calculate and assess environmental impact. Frameworks and assessments are being developed to help achieve this, which focus on questions around the types of platforms that the data is supported on, the energy consumption of these platforms, and how they are powered. The UK government’s *Greening government: sustainable technology strategy 2020*, for example, already sets out how government should conduct information and communication technology procurement in a sustainable way [22], and a cross-government Sustainable Technology Advice & Reporting Team, which coordinates digital sustainability work across academia, government, professional bodies and industry bodies [23], has developed various frameworks of best practice, that include how to assess environmental impacts.^1^ This not only provides useful guidance but also acts to increase awareness about the issues at stake. In another UK example, the digital health arena, NHSDigital has established a working group to look at developing a sustainable technology workstream for the UK National Health Service (NHS) [24,25], and has produced a sustainable development management plan that aims to identify, assess, raise awareness and reduce environmental impacts.^2^

However, calculations of environmental impacts are often complicated by technical and social difficulties. For example, we know from other sectors that as various actors attempt to calculate and cost the environmental impacts of ICT, they have been faced with inconsistent, sparse, and hard to compare data, and access to data [26]. This includes a lack of evidence-based guidance on how to make these calculations: different studies use different methodologies and so are difficult to compare. Furthermore, there is lack of consideration of the full supply chain of technology (from raw material to recycling). Moreover, the scale of data storage, data transfer and data processing are context dependent and so need to be considered differently and on a case-by-case basis. For example, smaller-volume actors who choose to procure commodity computing will have different difficulties in accessing information on their environmental costs than those actors who build their own data infrastructures. While it is increasingly possible to access some sustainability indication via companies who report on Ethics, Sustainability, and Governance (ESG) values, some of which account for an ICT footprint, assessments have been particularly complicated by a lack of shared understanding of ethics responsibility between public and private sectors. Private corporations are often unwilling to release data on energy consumption about these impacts, and because disclosure is voluntary, there are ‘few incentives for technology companies to release it’ [13,26]. While there is a move for corporations to become ‘greener’, in analogy to ‘ethics-washing’ [27,28] some consider these initiatives nothing more than ‘green-washing’ [26]. This lack of available tools to assess the environmental impact of open science initiatives takes up time and resources of any individual trying to identify this information, and this may be a limitation for researchers establishing open science frameworks.

## 3 Difficulties Enacting Responsibilities towards Environmentally Sustainable Open Science

A second key challenge relates to if individuals do calculate environmental impacts, they may come up against various obstacles regarding how to responsibly balance any calculated impacts (or estimates) against the social value of open science, and how much one should be prioritised over the other. For example, for those who have made the decision to procure data storage externally, decisions need to be made when procuring these facilities in terms of cheaper, less environmentally sustainable facilities versus those that are more expensive, but more environmentally aware. In certain instances, there may be no further resources or funding to allow the most environmentally appropriate choice to be made (this is especially the case if funders or research institutions do not act responsibly to provide such funding (see below)). These decisions might also need to include aspects of local or distributed storage. Other thorny decisions might include when and whether to allow data processing and re-use of open science data (if it has variable access options). Here, for example, we could ask the question, should data access be determined by how the data will be used, and only permit access if the processing of the data is deemed of sufficient value (and with sufficiently energy efficient methodologies) to offset the environmental cost? There are still little to no ethics governance frameworks or policy-inscribed standards of practice to assist with this decision-making, and few accountability measures to provide incentives to responsibly make these decisions.

Beyond the above, we also foresee a challenge relating to the infrastructure needed to enact environmentally sustainable open science ethical and social responsibilities. Acting responsibly in research institutions is complicated by the network within which responsible actors (i.e., actors wanting to act responsibly) are embedded. This network, or ethics ecosystem [29], includes interconnected actors existing at a number of levels, and at different points of the open science production process. This comprises individuals (researchers), organisations (research institutions and the various committees within), external bodies (publishing houses, funding bodies, professional associations and the governance policies they produce), and the for-profit sector (data storage and platform*/*service*/*tool providers). When the ethics ecosystem is acting properly, there is a shared understanding across all of these (and other) actors about how to act responsibly, and all actors participate equally in the promotion, evaluation and re-enforcement of this shared understanding of ethically responsible behaviour [29]. However, if there is inconsistent or little understanding about how to act responsibly (i.e., what “responsibility” looks like), and*/*or if these responsibilities are not shared, valued and*/*or practiced by all actors in the ecosystem, it becomes difficult for any particular actors (individual or organisational) to enact their own sense of responsibility [30].

These inconsistencies in the ethics ecosystem are evident when considering responsibilities attached to the environmental sustainability of the digital infrastructures of open science. If funding bodies responsibly promote open science, but choose not to enact a responsibility towards an environmentally sustainable open science by, for example, including a section on their funding calls for researchers to state explicitly how they will mitigate as much as possible the environmental impacts of their open science, it is unlikely that researchers will take on this responsibility. Equally, if journal editors act responsibly by, for example, providing infrastructures for open science publishing, but do not provide incentives for researchers to equally consider the environmental consequences of their open science databases*/*platforms (something that could be considered as enacting responsible best practice for an environmentally sustainable open science), then researchers are similarly less likely to also enact such responsibilities because such a requirement is not a sufficient gatekeeper for publishing their findings. The same holds for research institutions and professional bodies. Each can responsibly develop policies about the need to consider environmental impacts of open science, but challenges emerge in ensuring that the values enshrined within such policies are not only shared, but crucially enacted by all members of the ecosystem in a way that provides incentives and support for an environmentally sustainable digital infrastructure for open science. In these instances, while researchers and*/*or other actors may want to act responsibly, if the network to do so does not exist, and if the responsibility to do so is not enacted across the whole network, it makes it increasingly difficult for this desire to be responsible to be enacted [30]. Indeed, moving towards open science alone already requires a major change in the practice of many research communities, institutions and funders [2]; to make this already arduous move environmentally sustainable will be a complex, but not unsurmountable, mission to achieve.

## 4 Concluding Remarks

In highlighting these challenges, we call for all actors of the open science ethics ecosystem—including funding bodies, research institutions, scientific journals, professional institutions, those representing organisations or companies associated with the data infrastructure, etc.— to actively engage in discussions about how to move towards environmentally sustainable open data and science initiatives that take into account the environmental impact of data and digital infrastructures. Furthermore, any ethical framework that considers the environmental impacts of open science must fit within wider frameworks that consider the environmental sustainability of data science more generally. This would include not only an investment of resources in developing frameworks and tools that can help actors in the research ecosystem with the assessment of such environmental impacts, but also address the trade-offs in decisions that are made in this field, and data science more generally. For instance, choices relating to the energy provider, the database storage and*/*or platform provider (if applicable—we note here that a number of researchers who have smaller data volumes may use commodity computing to store and process their data rather than outsourcing this function), and the energy consumption of the data centre should all feature environmental impacts as an important element in the balancing act when making these decisions. Such decisions are, of course, complicated by the fact that the scale of data storage, data transfers and data processing change massively across the research ecosystem, and so the requirements may vary by actors. Finally, a system of support and incentives should be created to back more environmentally sustainable data infrastructures for not only open science projects but of data projects more generally.

## Footnotes

1
https://assets.publishing.service.gov.uk/government/uploads/system/uploads/attachment_data/file/837733/star-ict-annual-report-2018-2019a.pdf

2
https://digital.nhs.uk/about-nhs-digital/corporate-information-and-documents/sustainable-development-management-plan-summary-report-2017-2020
